# Chancen und Risiken der Telemedizin in der Urologie

**DOI:** 10.1007/s00120-022-01759-x

**Published:** 2022-02-07

**Authors:** Christian Wülfing

**Affiliations:** Chefarzt Urologie, Asklepios Klinik Altona, Paul-Ehrlich Str. 1, 22763 Hamburg, Deutschland

**Keywords:** Internet, Arzt-Patienten-Kontakt, Elektronische Patientenakte, Datenschutz, Videosprechstunde, Internet, Physician-patient relations, Electronic health records, Data protection, Remote consultation

## Abstract

Die Telemedizin in Deutschland ist auf dem Vormarsch. Die gesetzlichen Rahmenbedingungen für die Telemedizin sind seit 2018 sehr vorangetrieben worden. Neben dem Terminservice- und Versorgungsgesetz (TSVG) traten das Digitale-Versorgung-Gesetz (DVG) und das Patientendaten-Schutz-Gesetz (PDSG) sowie das Krankenhauszukunftsgesetz (KHZG) in Kraft. Auf der Anbieterseite kommen nun besonders die digitalen Gesundheitsanwendungen (DiGA), die zukünftig als „App auf Rezept“ verfügbar und von den Kostenträgern erstattbar sein werden, in den Markt (inklusive urologischer Anwendungen). Ebenso ist die Videosprechstunde nicht zuletzt durch die Coronapandemie sehr vorangekommen und dabei, sich weiter zu etablieren. Auch die elektronische Patientenakte (ePA) wird hoffentlich in hoher Geschwindigkeit umfassend verfügbar sein und genutzt werden, weil hierdurch ein starker Effizienzschub in der täglichen Arbeit eintreten wird. Dieses wird ergänzt durch das E‑Rezept und weitere Innovationen im Bereich der Medikamentenversorgung und -zustellung, die diese Entwicklung unterstützen werden. Als Herausforderungen im Umsetzungsprozess der Telemedizin bleiben v. a. die sehr wichtigen Aspekte des Datenschutzes, der Datensicherheit und die Problematik der Interoperabilität erhalten, werden hoffentlich aber den Fortschritt, der dringend benötigt wird, nicht sonderlich abbremsen. Urologinnen und Urologen werden durch die Fortschritte in der Digitalisierung der Medizin und der Urologie einen erheblichen Effizienzgewinn in der täglichen Arbeit erreichen.

## Abstract (en)

Use of telemedicine continues to increase in Germany. The legal framework has been expedited by several laws, such as the Appointment Service and Supply Act (TSVG), Digital Care Act (DVG), Patient Data Protection Act (PDSG) and Hospital Future Act (KHZG). The first digital health applications (DiGAs) that can be prescribed by doctors and will be reimbursed by medical insurance have entered the market and many more will follow, including urological applications. Video consultations have seen a remarkable increase driven by the Corona pandemic and will continue to be fully implemented in daily work. Electronic patient records will also be widely available within the next few years and will have a positive influence on efficiency in health care, as all steps in the treatment process will be digitalized. This will be supported by the e‑prescription and customer-friendly pathways of distribution of their medications. The most important challenges that hopefully do not inhibit the very much needed progress in the digitalization of healthcare are data protection and the compatibility between systems. Driven by the advances in digital healthcare and urology, urologists will experience a significant gain of efficiency in daily work.

## Hintergrund

Die rasanten Entwicklungen im Bereich des Internets, der digitalen Anwendungen und die damit einhergehenden wachsenden Bedürfnisse auf Seiten der Konsumenten haben in den letzten Jahren zu einem Einzug digitaler Technologien in praktisch allen Lebensbereichen geführt. Im Gesundheitsbereich scheint sich der Einzug der Telemedizin bei Ärzteschaft und Patienten allerdings schwer zu tun und hinter der Digitalisierungsgeschwindigkeit in anderen Lebensbereichen zurück zu bleiben. Was sind die Gründe hierfür? Besteht überhaupt Bedarf für Telemedizin? Wenn ja, gibt es genügend und auch bedarfsgerechte Angebote? Hat die Coronapandemie zu einem Umdenken und einem Schub der Telemedizin geführt? Oder überwiegen die Hemmnisse, weil Regulation, Skepsis und Bürokratie die eigentlich sinnvollen und gewünschten Entwicklungen aufhalten?

Der Einzug der Telemedizin bei Ärzteschaft und Patienten scheint sich schwer zu tun

Dieser Artikel soll eine Bestandsaufnahme zur Telemedizin darstellen und insbesondere Mut machen, die Chancen des bevorstehenden Wandels zu erkennen und auch in der Urologie zu nutzen.

## Definition und aktueller Stand der „Telemedizin“ in Deutschland

Die im englischen (und deutschen) Sprachgebrauch durchaus übliche Bezeichnung „Digital Health“ umfasst laut „Wikipedia“ „… die interdisziplinäre Verbindung von Gesundheit, Gesundheitsfürsorge, Leben und Gesellschaft mit digitalen Medizin- und Gesundheitstechnologien, um die Effizienz der Gesundheitsversorgung zu verbessern und Arzneimittel individueller und wirkungsvoller einsetzen zu können“ [1]. Die gesetzlichen Rahmenbedingungen für die Telemedizin sind seit 2018 sehr vorangetrieben worden. Neben dem Terminservice- und Versorgungsgesetz (TSVG) traten das Digitale-Versorgung-Gesetz (DVG) und das Patientendaten-Schutz-Gesetz (PDSG) sowie das Krankenhauszukunftsgesetz (KHZG) in Kraft.

**Figure 1:**
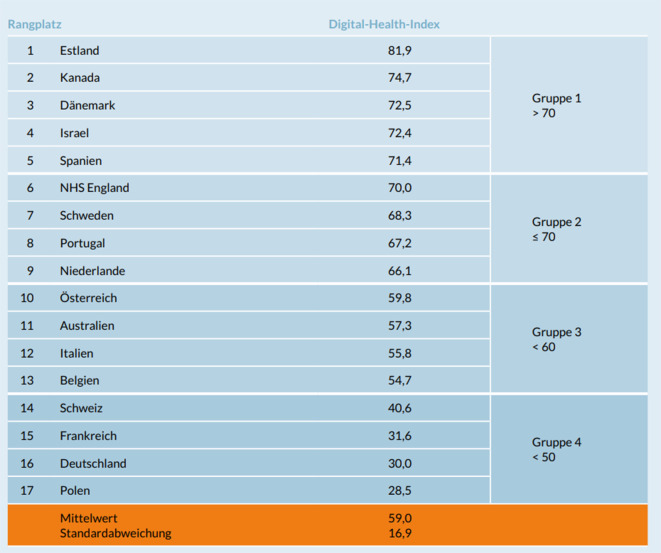

Zum aktuellen Stand der Entwicklung von „Digital Health“ in Europa wurde kürzlich von der Bertelsmann Stiftung die Studie #SmartHealthSystems beauftragt [2]. Hier wurde explizit der Frage nachgegangen warum Deutschland im internationalen Vergleich zu anderen Nationen bei der Digitalisierung im Gesundheitswesen noch deutlich hinterher hinkt und wie diejenigen Erfolgsfaktoren identifiziert werden können, die für einen Fortschritt zur Verbreitung von „Digital Health“ sorgen. In dieser Studie wurden insgesamt 17 Länder aus der EU und der OECD hinsichtlich 34 verschiedener Indikatoren aus den Feldern „Policy-Aktivität“ (politisch-strategisches Vorgehen), „digital health readiness“ (technische Implementierung und technischer Reifegrad) und der tatsächlichen Datennutzung analysiert. Die ernüchternde Erkenntnis dieser Studie war, dass Deutschland im Ranking des aus den genannten Sub-Indizes zusammengesetzten Digital Healt Index den vorletzten Platz belegte (Abb. 1).

Es zeigt sich ein klarer Trend zur Videosprechstunde

Eine weitere wichtige Standortbeschreibung zum Thema lässt sich aus dem „eHealth Monitor 2020 Deutschlands Weg in die digitale Gesundheitsversorgung – Status quo und Perspektiven“ der Beratungsgesellschaft McKinsey ablesen [3].

Die in diesem Bericht genannten Fakten geben gleichermaßen Anlass zu Sorge und Optimismus: Im Jahre 2019 boten noch immer 59 % der ambulanten Ärzte und Psychotherapeuten ihren Patienten keinerlei administrativen und digitalen Gesundheitsservices an. Im Jahre 2018 waren dies 61 % gewesen. Auf der anderen Seite konnte ein klarer Trend für die Videosprechstunde gesehen werden: während dies in 2017 nur von 2 % der Ärzte angeboten wurde, betrug die Quote (vermutlich unterstützt durch die Coronapandemie) bereits 52 %. Auch auf Seiten der Anwender (Patienten) zeigten sich interessante Entwicklungen: Bereits 59 % der der Teilnehmer einer Umfrage können sich aktuell bereits vorstellen, verschreibungspflichtige Apps zu nutzen.

## Chancen der Telemedizin in der Urologie

Der Begriff Telemedizin umfasst eine Vielzahl von digitalen Anwendungen, von Hardware- und Softwarelösungen, web-basierten Diagnosen, digitalen Gesundheits-Trackern und Überwachungstools. Diese werden zu einem verbesserten Nutzen und zu einer Verbesserung der Chancen gleichermaßen bei Patientinnen und Patienten und bei der Ärzteschaft führen. Auf diese Weise wird die digitale Medizin neue Wege erschließen und die Effizienz des Gesundheitswesens steigern. Im Folgenden werden unterschiedliche Anwendungen und Aspekte der Telemedizin vorgestellt und die Chancen aufgezeigt:

### Online-Terminierung

Von Notfällen abgesehen beginnt die Mehrheit der Patienten-Arzt-Kontakte traditionell mit der Vereinbarung eines Termins. Zahlreiche Anbieter sind in den letzten 2–3 Jahren in den Markt eingetreten und bieten diese Services an. Der Autor dieses Artikels weiß selbst nur zu gut, wie schön es ist, dem hilfesuchenden Patienten von Anfang an „ein gutes Gefühl“ zu geben, in dem die empathischste Mitarbeiterin geduldig mit ihm oder ihr einen Termin beim Arzt der Praxis oder Klinik vereinbart. Aber seien wir ehrlich: Zum einen steigt auf Patienten- (Kunden‑)Seite stetig die Nachfrage nach digitalen Anwendungen und Terminvereinbarungsmöglichkeiten. Schließlich nutzt man ja auch für Kino, Konzert, Restaurant etc. zunehmend elektronische Tools, um zum erwünschten Platz zu kommen. Zum anderen ist es kein Geheimnis, dass die konventionellen Terminvereinbarungsgespräche in hohem Maße die Personalressource belasten und enorm viel Zeit (und Geld) kosten. Daher liegt die Chance darin, dass zumindest denjenigen Patientinnen und Patienten, die über eine Affinität zu digitalen Medien verfügen und vielleicht sogar aktiv nach einer elektronischen Möglichkeit der Terminvereinbarung suchen, ein solcher Service angeboten wird, um die Gesamtadministration der Praxis prozessual und betriebswirtschaftlich zumindest um diesen Patientenanteil zu entlasten.

### Digitale Gesundheitsanwendungen (DiGA)

Die DiGA wurden mit dem DVG ins Leben gerufen. Sie sind digitale Medizinprodukte, die von Patientinnen und Patienten als „App auf Rezept“ geladen und für die unterschiedlichsten Gesundheitsthemen genutzt werden können. Von den DiGA sollen positive Versorgungseffekte für die Medizin ausgehen: so können entweder der Nachweis eines medizinischen Nutzens (Verbesserung von Morbidität, Mortalität oder Lebensqualität) und/oder patientenrelevante Struktur- und Verfahrensverbesserungen erreicht werden. Zugelassene DiGA sollen zukünftig regelhaft von den Krankenkassen erstattet werden.

Von den DiGA sollen positive Versorgungseffekte für die Medizin ausgehen

Die Liste der bereits zugelassenen DiGA umfasst bislang Anwendungen aus den Bereichen Diabetes, Psychosomatik und Psychiatrie, Onkologie etc. Konkrete Anwendungen für die Urologie sind die App „Kranus Edera“ (Kranus Health GmbH) zur Behandlung der erektilen Dysfunktion und die App „Mika“ (Fosanis GmbH) die für uroonkologische Erkrankungen auch von Urologinnen und Urologen heute schon verschrieben werden kann. Weitere DiGA aus den Bereichen Prostatakarzinom, Inkontinenz („Uroletics“, Rocketlane Medical Ventures GmbH) u. a. sind in Vorbereitung.

### Videosprechstunde

Die Coronapandemie hat das Phänomen Videosprechstunde deutlich vorangetrieben. Während vor der Pandemie ca. 3000 Videosprechstunden in Deutschland abgehalten wurden, waren es im ersten Halbjahr 2020 schon 1,4 Mio. Videosprechstunden [4]. Der rechtliche Rahmen ist mit der Liberalisierung des Fernbehandlungsverbots geschaffen worden. Die Abrechnungsmöglichkeiten insbesondere im GKV-Bereich sind zwar verbessert worden, erscheinen aber noch ausbaufähig. Auf der technischen Seite sind zahlreiche Anbieter im Markt vertreten, so dass die konkrete Einrichtung realistisch und leicht umsetzbar erscheint. Welche Rolle wird die Videosprechstunde (auch über die Coronapandemie hinaus) zukünftig spielen? Wie könnte ein idealer Einsatz dieser digitalen Sprechstundenmöglichkeit aussehen? Aus Sicht des Autors ist es notwendig, das Konzept der Videosprechstunde genau zu durchdenken. Vermutlich ist es nicht sinnvoll, eine Videosprechstunde genau wie einen normalen Sprechstundentermin „nur per Video“ abzuhalten. Vielmehr müsste überlegt und entwickelt werden, welche Situationen sich im urologischen Sprechstundenalltag für eine Videokonsultation anbieten. Der Videotermin sollte im Hinblick auf die Wirtschaftlichkeit kurz und effizient vorbereitet sein. Hierzu wäre es wünschenswert, dass die telemedizinischen Anbieter nicht nur eine reine Videoplattform (ähnlich wie „FaceTime“ oder „Skype“) anbieten, sondern der Videotermin durch Bereitstellung von elektronischen Fragebögen etc. begleitet wird, so dass die eigentliche Videosprechstunde fokussiert über anderweitig digital eingespeiste Daten des Patienten stattfindet und somit deutlich zeiteffizienter eingesetzt werden kann. Man stelle sich vor, dass es flächendeckend zur Einführung der elektronischen Patientenakte kommen würde (s. unten). Ab diesem Moment würden die medizinischen Informationen schon vor dem eigentlichen Videotermin zur Verfügung stehen, so dass die Videosprechstunde hiermit deutlich sinnvoller und zeiteffizienter werden würde. Die Diskussionen um eine zu schlechte Bezahlung werden dann auch in einem anderen Licht stehen, weil mehr Videotermine pro Zeit realisiert werden könnten.

### Elektronische Patientenakte (ePA)

Wenn man das Thema „ePA“ optimistisch sehen möchte, dann könnte man sagen, „endlich geht es los … !“ Der Gesetzgeber hat die wesentlichen juristischen Rahmenbedingungen für die Digitalisierung des Gesundheitssystems gelegt. Mit dem Jahr 2021 wurde ein straffer, verbindlicher Zeitplan gestartet und das TSVG hat die Krankenkassen verpflichtet, den Versicherten eine ePA anzubieten. Während die Benutzung der ePA ursprünglich im Sinne einer freiwilligen Entscheidung für Patienten geplant war, hat die neue Bundesregierung im Koalitionsvertrag im Dezember 2021 festgelegt, dass alle Patienten verpflichtend eine ePA bekommen sollen, hat ihnen aber im Sinne der Freiwilligkeit eine „Opt-out“-Möglichkeit, diese nicht nutzen zu müssen eingeräumt, ein „Opt-out“ (Patient entscheidet, dass er die angebotene ePA nicht nutzt) statt ein „Opt-in“ (Patient entscheidet, ob er eine ePA nutzen möchte) ist eine sehr weise Entscheidung der neuen Regierung, denn warum sollte ein Patient aktiv nach der ePA fragen, wenn das gesamte System keine Incentivierung für die Verbreitung dieser an sich ja sehr positiven Idee vorhält.

Eine automatische Zuweisung einer ePA lässt also auch auf eine bessere Verbreitung und Akzeptanz hoffen, als wenn man auf die Freiwilligkeit auf Seiten der Patienten gesetzt hätte. So schreibt die Bertelsmann-Stiftung: „… Auch wenn die Weichen für die ePA gestellt sind, hängt der Erfolg letztlich von einer hohen Nutzung mit Mehrwert für Patienten und medizinisches Personal ab …“ [2].

Wir sind uns einig: Hätten wir über Nacht eine flächendeckende Ausstattung der Patientinnen und Patienten mit der ePA, würde das gesamte System einen deutlichen Effizienzschub bekommen, weil alleine die zahlreichen Übertragungswege (Telefon, E‑Mail und – ja, immer noch sehr oft: das Fax … !) und die damit verbundenen massiven Zeitaufwendungen deutlich schlanker werden. Allein dieser Zeitgewinn würde der gesamten Ärzteschaft ein dringendes „Aufatmen“ bescheren. Der Autor möchte daher im Sinne der Chancen dazu aufrufen, dass Urologinnen und Urologen eine sehr aktive Rolle beim Gestalten der nächsten Schritte der ePA übernehmen: Schaffen wir die Möglichkeiten und Anreize, dass Patientinnen und Patienten uns ihre Daten per ePA zur Verfügung stellen.

### E-Rezept und Versandapotheke

**Figure 2:**
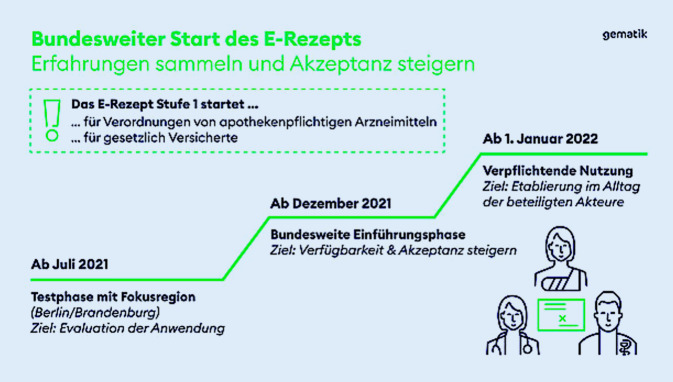

Ein wichtiger und originärer Bestandteil einer Telemedizin ist auch für Urologinnen und Urologen das Rezeptierungswesen und die Versorgung der urologischen Patientinnen und Patienten mit Medikamenten. Die Einführung des E‑Rezepts wurde bereits 2019 mit dem Gesetz für mehr Sicherheit in der Arzneimittelversorgung (GSAV) geregelt. In einer Testphase in der Metropolregion Berlin-Brandenburg wurde seit 01.07.2021 der offizielle und gesetzlich verpflichtende Start des E‑Rezepts zum 1. Januar 2022 vorbereitet (Abb. 2). Die konkrete Ausstellung des E‑Rezepts erfolgt über einen Rezeptcode, der digital erzeugt wird. Die Einlösung des E‑Rezeptes auf Seiten der Patientinnen und Patienten wird sodann über die E‑Rezept-App der Gematik GmbH abgewickelt. Ein E‑Rezept kann somit zukünftig über einen sicheren Zugang zu den Rezeptdaten in jeder Apotheke eingelöst werden.

Auch Versandapotheken werden durch das Voranschreiten der Digitalisierung wohl auch vermehrt in den Markt eintreten. Hier werden neben gesetzlichen Strukturen sicherlich auch die Patientinnen und Patienten als „Kunde“ die Nachfrage mitgestalten und selbst entscheiden, ob sie – wie traditionell üblich und erforderlich – in eine oder gar „seine/ihre “ Apotheke geht, oder aber das Medikament über eine Versandmöglichkeit zustellen lässt und somit auch diese Vorzüge der Telemedizin nutzt. Schon heute haben sich große Versandapotheken-Ketten etabliert (DocMorris, Zur Rose-Gruppe), die den Versand von Medikamenten zentral für Deutschland organisieren können, aber auch die „Apotheken vor Ort“, die als solche inzwischen zu einem eigenen Verband zusammengeschlossen sind (Pro AVO), bieten lokale Versandmöglichkeiten im entsprechenden regionalen Umkreis an.

Die konkrete Ausstellung des E‑Rezepts erfolgt über einen digital erzeugten Rezeptcode

Zusammenfassend wird das E‑Rezept nicht nur die Arzneimitteltherapiesicherheit vorantreiben, sondern den Anwendern (Urologinnen und Urologen!) hier auch durch verbesserte Abläufe enorm Zeit und Ressourcen einsparen.

### Telemedizinische Online-Plattformen und -Kliniken

TeleClinic, Kry, GoSpring, Zava etc. sind Schöpfungen im Bereich der Telemedizin, die in den letzten Jahren auf den Markt gekommen sind und sich einer hohen Beliebtheit bei den „Kunden“ erfreuen. Nicht nur die Möglichkeit, Diagnosen zu ermitteln (Ada Health), Videosprechstunden im großen Stil anzubieten (TeleClinic, Kry), sondern auch die Möglichkeit für unterschiedliche Symptome Diagnosen auf der Basis von intelligenten medizinischen Online-Fragebögen zu stellen und Medikamente online zu bestellen (Zava, GoSpring) hat sich im Markt etabliert.

Alle diese Online-Angebote haben gemeinsam, dass sie mit erheblichem Kapitaleinsatz gegründet und finanziert wurden. Sie werden von Menschen in hohem Maße nachgefragt. Sie arbeiten mit und generieren Daten. Die ersten Online-Plattformen haben durch diesen Ansatz einen interessanten Beitrag zur urologischen Versorgungsforschung beigetragen: So GoSpring (www.gospring.de), eine Marke der Wellster Healthtech GmbH aus München, die in 2 publizierten Studien über die innerhalb von 2 Jahren behandelten 80.000(!) Patienten mit erektiler Dysfunktion (ED) nicht nur darlegen konnten, warum Patienten den reinen Online-Weg wählen (Tab. 1), sondern die erstmals auch für bestimmte Subgruppen der ED-Patienten zeigen konnten, für die Tadalafil Sildenafil überlegen ist [6]. Durch diesen Ansatz, der durch das immense Datenaufkommen beliebig erweiterbar ist, könnten tatsächlich neue Dimensionen für die Versorgungsforschung, auch in der Urologie, entstehen.

**Table 1:** 

Warum nutzen Männer eine Online-Plattform zur Behandlung der ED?
48 % der Befragten führen ihre Behandlungsbarriere auf Bequemlichkeit hin
36 % der Befragten sagen, dass sie aus Scham und Diskretion vorher noch nie beim Hausarzt oder Urologen waren
42 % der Befragten, die bereits Kontakt mit Potenzmitteln hatten, haben sich diese über unsichere Zugangswege, wie den Schwarzmarkt, oder unseriöse Internetseiten besorgt

Nun stellt sich die Frage, ob diese Online-Plattformen als Konkurrenz für die Arztpraxen zu sehen sind? Vermutlich ja, denn jeder Patient, der sich online behandeln lässt, „fehlt“ der „echten“ Arztpraxis. Allerdings handelt es sich hier wohl häufig um Patienten, die ohnehin nicht zum „echten“ Arzt gehen würden, weil sie Gesundheitsdienstleistungen möglichst vollständig online abwickeln wollen („bloß nicht im Wartezimmer sitzen …“) und die sich in vielen Fällen aus medizinischer Sicht wahrscheinlich auch für eine Online-Behandlung eignen und die die bestehenden Patientenaufkommen in den Praxen ohnehin nur noch weiter eskalieren würden. Mit anderen Worten: Vielleicht sollen gar nicht alle diese Patienten zusätzlich in die Praxen kommen. Insofern hier die Chance: Könnten Online-Plattformen und -Kliniken zukünftig eine sinnvolle Ergänzung für Urologinnen und Urologen in den Praxen sein, in dem Sie durch Kooperationen oder auch eigenständige Online-Angebote die Patientinnen und Patienten teils online, teils in physischer Präsenz in der Praxis behandelt werden? Sicher bietet es sich für Urologinnen und Urologen an, diese „Hybrid-online-Zukunft“ aktiv mit zu gestalten, denn es wird bei diesen Angeboten sehr damit zu rechnen sein, dass sie im Markt bestehen bleiben und hier große Chancen bestehen für unsere Berufsgruppe.

## Risiken der Telemedizin in der Urologie

Bei aller Euphorie und Betonung der Chancen eines digitalmedizinischen Wandels: Es gibt auch Risiken und noch viele Hürden zu meistern. Die Hauptherausforderungen sind der Datenschutz, die Datensicherheit und eine Interoperabilität der Systeme.

Zukünftige Anbieter von DiGA und auch der ePA sind verpflichtet, die MIO zu nutzen

In der Telemedizin gelten grundsätzlich alle datenschutzrechtlichen Vorschriften, die auch sonst in der Medizin gültig sind. Gesundheitsdaten werden von der Datenschutzgrundverordnung (DSGVO) als besonderer Kategorie personenbezogener Daten angesehen und unterliegen damit einem besonderen Schutz. Hinzu kommen Datenschutzvorgaben bzgl. Online-Kommunikation und Datenweitergabe. Die in der Telemedizin üblichen zwei Arten von Datenübertragung, nämlich Datenaustausch zwischen Arzt und Patient auf der einen und Datenaustausch zwischen Arzt und Gesundheitseinrichtung zum anderen. Die Weitergabe der Gesundheitsdaten an Dritte ist nur mit einer entsprechenden Einwilligungserklärung des Patienten zulässig. Die Datensicherheit bezeichnet die generelle Sicherheit von Daten. Im besten Falle gewährleistet die Datensicherheit die Vertraulichkeit, die Integrität und die Verfügbarkeit von Daten.

Eine entscheidende Voraussetzung für die systemübergreifende Übertragung von Daten sind Standards, die eine Interoperabilität sicherstellen. Dies gilt für den technischen Bereich (Serverarchitektur und Schnittstellen), aber auch für den inhaltlichen Bereich (einheitliche Verwendung von Terminologie). Derzeit wird durch die Kassenärztliche Bundesvereinigung (KBV) eine Liste Medizinischer Informationsobjekte (MIO) erarbeitet, die zum Ziel hat, bundesweit standardisierte Formate für die Speicherung und den Austausch medizinischer Daten zu etablieren. Zukünftige Anbieter von DiGA und auch die ePA sind verpflichtet, diese MIO zu nutzen und den Export von Daten in z. B. die ePA zu realisieren.

Verschiedene Player im Gesundheitswesen haben kürzlich ein gemeinsames Konzeptpapier „Interoperabilität 2025“ vorbereitet und dem Bundesgesundheitsministerium vorgelegt [8]. Das dort vorgelegte Konzept beschreibt eine zielführende Umsetzung einer Strategie, an deren Ende die Interoperabilität der Gesundheitsdaten in Deutschland erreicht wird.

## Fazit für die Praxis

- Die Telemedizin in Deutschland ist nicht mehr aufzuhalten. Wenngleich das Tempo der Umsetzung langsam erscheint, müssen dennoch viele Weichen gestellt werden, die der Integration digitaler Anwendungen Vorschub leisten werden.
- Auch der Fachbereich der Urologie wird aus dieser Entwicklung viele Chancen generieren können. Ob es neue digitale Gesundheitsanwendungen (DiGA), die Umsetzung der elektronischen Patientenakte (ePA) oder die Implementierung der Videosprechstunde und des E‑Rezepts sind.
- Die Urologinnen und Urologen werden aus den Neuerungen der Telemedizin viele Möglichkeiten zu Effizienzsteigerung der täglichen Arbeit finden. Diese sollten schnell und effizient genutzt werden.
